# Commentary: Incorporation of membrane-anchored flagellin or *Escherichia coli* heat-labile enterotoxin B subunit enhances the immunogenicity of rabies virus-like particles in mice and dogs

**DOI:** 10.3389/fmicb.2015.01039

**Published:** 2015-09-29

**Authors:** Arun Kumar

**Affiliations:** T-cell Platform, Translational Medicine, Novartis Vaccines and Diagnostics (a GSK Company)Siena, Italy

**Keywords:** rabies, VLP, vaccines, recombinant proteins, viruses

Rabies virus is accountable for substantial economic burden, and possesses high risk to the individuals living in low-income countries. Rabies is fatal encephalitis caused by the rabies virus (RABV) and usually transmit to humans through bites from rabid animals. The causative agent for this life threatening disease is RABV, containing single-stranded negative-sense RNA as genetic material (Jackson, 2013). Annually, rabies is responsible for approximately 55,000 deaths worldwide (a substantial part of deaths occur in developing countries); (Ge et al., 2011). Vaccination is the principal path for prevention and control of the disease. Currently available cell culture based rabies vaccines possess several side effects (e.g. fever, headache, neurological disease etc.) and high cost in developing countries usually require many doses to provide pre and post-exposure protection against the RABV. Several, improved vaccines based on anti-rabies monoclonal antibodies, lyophilized, and cell culture technology, are already under clinical development phase (Kaur et al., 2015). Furthermore, recombinant molecules have also been used as vaccine candidates very extensively. Further, immunogenicity of these molecules can be enhanced by combining adjuvants and immuno-stimulatory molecules. In the past, Wen et al. demonstrated that recombinant rabies virus (rRABV) expressing dendritic cell activating molecules such as granulocyte-macrophage colony stimulating factor (GM-CSF), macrophage-derived chemokine (MDC), and macrophage inflammatory protein (MIP-1a), could enhance RABV immunogenicity (Wen et al., 2011). Moreover, Zhou et al. suggested that rRABV expressing bacterial flagellin could also induce robust adaptive immune responses (Zhou et al., 2013). Flagellin is a very effective and potent adjuvant because of non-inducer of IgE production and at very low dose (1–10 μg in non-human primates) very efficient to induce effective immune responses (Mizel and Bates, 2010). Recombinant virus-like particles (VLPs) are promising vaccine candidates, because they mimic original virus structure and are able to induce potent humoral and cellular immune responses (Kumar et al., 2011). Moreover, VLP possesses several advantages over the conventional vaccines because of the lack of genetic material, stable in extreme environment and capable of expressing foreign molecules. Very recently, Fontana et al. (2014) showed that VLPs containing glycoprotein of RABV are very efficient in induction of antibody mediated immune responses in Balb/c mice. Qi et al. (2015) combined all these novel strategies, in order to develop an effective RABV vaccine. In present report, Qi and colleagues constructed chimeric RABV-like particles (cRVLPs) containing RABV glycoprotein and matrix protein, expressing membrane-anchored flagellin or E. coli heat-labile enterotoxin B (LTB). Immunological studies in BALB/c mice and dog models demonstrated that both humoral and cellular immune responses were stronger with cRVLPs, compared with standard rabies VLPs (sVLPs). Further, Qi et al. (2015) suggest that flagellin promote antigen processing and presentation by inducing higher expression of MHC I and II on the surface of DCs. Interestingly, in a pioneer study, mucosal delivery of inactivated influenza vaccine have been shown to induce heterotypic immunity against H1N1 virus, when administered with heat-labile enterotoxin B (Tumpey et al., 2001). These attributes make flagellin and enterotoxin B outstanding adjuvants for use in vaccines.

In conclusion, present and previous reports suggest that chimeric VLPs are novel class of subunit vaccines and possess capability to induce both protective innate and adaptive immune responses. Possibly, use of immuno-modulators along with cRVLPS may enhance the efficacy of existing rabies vaccines. Further, detailed mechanism of the comprehensive effect of flagellin and enterotoxin B stimulated T and B-cells, on antiviral immune responses, should be investigated.

## Conflict of interest statement

The author declares that the research was conducted in the absence of any commercial or financial relationships that could be construed as a potential conflict of interest.

## References

[B1] Fontana D. Kratje R. Etcheverrigaray M. Prieto C. (2014). Rabies virus-like particles expressed in HEK293 cells. Vaccine 32, 2799–2804. 10.1016/j.vaccine.2014.02.031 24631077

[B2] Ge J. Wang X. Tao L. Wen Z. Feng N. Yang S.. (2011). Newcastle disease virus-vectored rabies vaccine is safe, highly immunogenic, and provides long-lasting protection in dogs and cats. J. Virol. 85, 8241–8252. 10.1128/JVI.00519-11 21632762 PMC3147977

[B3] Jackson A. C. (2013). Current and future approaches to the therapy of human rabies. Antiviral Res. 99, 61–67. 10.1016/j.antiviral.2013.01.003 23369672

[B4] Kaur M. Garg R. Singh S. Bhatnagar R. (2015). Rabies vaccines: where do we stand, where are we heading? Expert Rev. Vaccines 14, 369–381. 10.1586/14760584.2015.973403 25348036

[B5] Kumar A. Chen T. Pakkanen S. Kantele A. Söderlund-Venermo M. Hedman K.. (2011). T-helper cell-mediated proliferation and cytokine responses against recombinant Merkel cell polyomavirus-like particles. PLoS ONE 6:e25751. 10.1371/journal.pone.0025751 21991346 PMC3185038

[B6] Mizel S. B. Bates J. T. (2010). Flagellin as an adjuvant: cellular mechanisms and potential. J. Immunol. 185, 5677–5682. 10.4049/jimmunol.1002156 21048152 PMC3756556

[B7] Qi Y. Kang H. Zheng X. Wang H. Gao Y. Yang S.. (2015). Incorporation of membrane-anchored flagellin or *Escherichia coli* heat-labile enterotoxin B subunit enhances the immunogenicity of rabies virus-like particles in mice and dogs. Front. Microbiol. 6:169. 10.3389/fmicb.2015.00169 25784906 PMC4347500

[B8] Tumpey T. M. Renshaw M. Clements J. D. Katz J. M. (2001). Mucosal delivery of inactivated influenza vaccine induces B-cell-dependent heterosubtypic cross-protection against lethal influenza A H5N1 virus infection. J. Virol. 75, 5141–5150. 10.1128/JVI.75.11.5141-5150.2001 11333895 PMC114919

[B9] Wen Y. Wang H. Wu H. Yang F. Tripp R. A. Hogan R. J.. (2011). Rabies virus expressing dendritic cell-activating molecules enhances the innate and adaptive immune response to vaccination. J. Virol. 85, 1634–1644. 10.1128/JVI.01552-10 21106736 PMC3028913

[B10] Zhou M. Zhang G. Ren G. Gnanadurai C. W. Li Z. Chai Q.. (2013). Recombinant rabies viruses expressing GM-CSF or flagellin are effective vaccines for both intramuscular and oral immunizations. PLoS ONE 8:e63384. 10.1371/journal.pone.0063384 23700422 PMC3658976

